# Wafer-scale growth of VO_2_ thin films using a combinatorial approach

**DOI:** 10.1038/ncomms9475

**Published:** 2015-10-09

**Authors:** Hai-Tian Zhang, Lei Zhang, Debangshu Mukherjee, Yuan-Xia Zheng, Ryan C. Haislmaier, Nasim Alem, Roman Engel-Herbert

**Affiliations:** 1 Department of Materials Science and Engineering and Materials Research Institute, Pennsylvania State University, University Park, Pennsylvania 16802, USA; 2 Department of Physics, Pennsylvania State University, University Park, Pennsylvania 16802, USA

## Abstract

Transition metal oxides offer functional properties beyond conventional semiconductors. Bridging the gap between the fundamental research frontier in oxide electronics and their realization in commercial devices demands a wafer-scale growth approach for high-quality transition metal oxide thin films. Such a method requires excellent control over the transition metal valence state to avoid performance deterioration, which has been proved challenging. Here we present a scalable growth approach that enables a precise valence state control. By creating an oxygen activity gradient across the wafer, a continuous valence state library is established to directly identify the optimal growth condition. Single-crystalline VO_2_ thin films have been grown on wafer scale, exhibiting more than four orders of magnitude change in resistivity across the metal-to-insulator transition. It is demonstrated that ‘electronic grade' transition metal oxide films can be realized on a large scale using a combinatorial growth approach, which can be extended to other multivalent oxide systems.

## Abstract (web summary)

Precise valence state control to avoid performance deterioration in transition metal oxide films has proved challenging. Here, the authors establish a combinatorial approach to create a valence state library of VO_2_, allowing for the growth of wafer size VO_2_ thin films.

## Graphical abstract

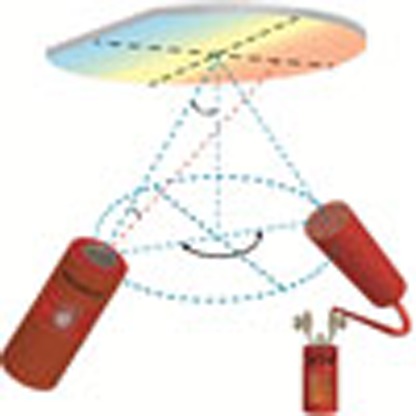

Multivalent transition metal oxides exhibit a wealth of interesting and useful properties that are beyond conventional semiconductors commonly employed in electronic and optoelectronic devices^1^,^2^,^3^,^4^. The energetically preferred ground state in this class of material is dominated by the valence state of the transition metal cations, giving rise to different types of order in the spin, charge or orbital degree of freedom^5^,^6^,^7^. Utilizing these functionalities to realize a new generation of electronic and optoelectronic devices requires manufacturing of transition metal oxide films with superior quality on a wafer scale. However, this has been proved challenging because film properties are highly sensitive to valence state variations, which can easily get compromised if the synthesis approach is plagued by an inferior valence state control^5^,^6^,^8^,^9^,^10^.

The commonly employed strategy towards stoichiometric multivalent oxide thin films involves extensive calibration series, whereby relevant growth parameters are systematically varied to identify optimal growth condition. In most cases, this is a daunting task, involving tedious and costly calibration series, which sometimes become necessary even in the favourable case of self-regulated growth of ternary transition metal thin films^11^,^12^,^13^, since it only addresses cation stoichiometry, leaving the determination of the demanded cation-to-anion ratio unaddressed.

The challenge is also present in multivalent binary oxides. VO_2_ has recently drawn much interest due to its electronic phase transformation from a metal to an insulator near room temperature^14^,^15^, making it attractive for next-generation transistors^16^, non-Boolean computing^17^ and memory metamaterials^18^. Recently, metamaterial electric circuits have also been demonstrated based on high-quality VO_2_ film grown on silicon wafer, which has potential application in adaptive matching resistor networks^19^. Although phase pure epitaxial VO_2_ films have been grown by pulsed laser deposition (PLD)^20^,^21^,^22^, sputtering^23^,^24^,^25^, molecular beam epitaxy (MBE)^26^,^27^ and chemical solution deposition technique^28^, the growth of high-quality VO_2_ thin films has been found very demanding^21^,^26^, attributed to the complex and rich structural phase diagram of vanadium oxide^5^. Vanadium oxide phases with lower or higher oxidation states than the targeted VO_2_ phase, namely, V_*n*_O_2*n*−1_ (Magnéli phases)^29^ or V_*n*_O_2*n*+1_ (Wadsley phases)^5^, can easily form if the vanadium to oxygen ratio is not precisely controlled throughout the growth^21^,^26^. The lack of valence state control directly compromises the metal-to-insulator transition (MIT), significantly reducing the resistivity change across the MIT^8^,^30^.

One possible strategy to address the valence state control during growth is the application of a combinatorial approach^31^,^32^,^33^,^34^,^35^,^36^,^37^, where a flux gradient across the sample is intentionally introduced during film growth to generate various growth conditions simultaneously at different sample locations. Film properties within the composition library are then characterized and related to the flux ratio calculated from the geometrical arrangement of sources and the sample. Such a combinatorial strategy requires (1) precise and direct control of a spatially varying fluxes; (2) two or more sources to independently supply the elements of interest; (3) a favourable deposition geometry, that is, a sufficiently large substrate compared with the flux density profile at sample position to create a sufficiently steep flux gradient; (4) large inelastic mean free path to avoid unintentional gas phase reactions. This approach has been successfully utilized to optimize the bandgap of III–V semiconductor nanowires^38^, discover new luminescent materials^33^, study the composition variance of metallic glasses^34^, increasing the Curie temperature of the ferromagnetic semiconductor GaMnAs^37^ and exploring the effect of LaAlO_3_ cation stoichiometry on electrical properties of the two-dimensional electron gas at the LaAlO_3_/SrTiO_3_ interface^35^.

Extending the combinatorial approach to binary oxides requires the supply of oxygen in a spatially varying fashion, which seems impractical in PLD, sputtering or MBE, where oxygen is supplied as a gas. Considering the large oxygen pressures (∼10 mtorr)^21^ required to stabilize stoichiometric VO_2_, the small inelastic mean free path of <1 cm severely limits the magnitude of oxygen concentration gradients across the wafer, rendering the combinatorial approach inapplicable using traditional growth techniques. We have circumvented this difficulty by supplying oxygen in a form pre-bonded to vanadium, which can be used to generate an oxygen concentration gradient across the wafer, thus locally varying the oxygen activity. For the oxygen-containing precursor molecule vanadium oxytriisopropoxide (VTIP), each vanadium atom is in the valence state 5+, forming three single bonds and one double bond to oxygen. Purely supplying VTIP with a V:O ratio of 1:4 resulted in an oxygen surplus in the film, which was compensated by independently supplying vanadium metal. Using this approach, a VO_2_ valence state library was deposited on a 3-inch *r* plane sapphire wafer and the optimal growth condition was identified within a single film growth. Under the optimized growth condition, single-crystalline VO_2_ films of superior quality were grown epitaxially on wafer-scale areas with excellent uniformity, exhibiting more than four orders of magnitude resistivity change across the MIT, being among the highest values reported for thin films.

## Results

### Combinatorial growth of VO_2_ thin films

**Figure 1:**
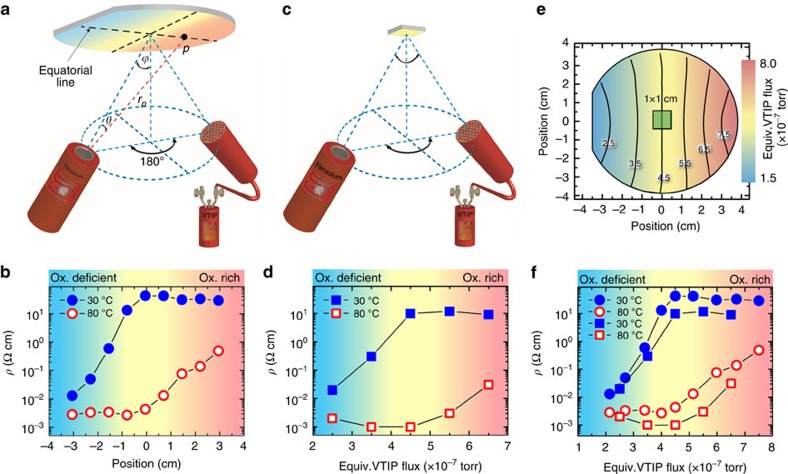
Combinatorial growth method. (**a**) Schematic of V-effusion cell, VTIP gas injector and a 3-inch wafer arrangement to create a gradient of VTIP:V flux ratios along the equatorial line across the wafer. (**b**) Resistivity at 30 (insulating state) and 80 °C (metallic state) measured from nine devices of VO_2_ on *r*-Al_2_O_3_ located at different positions on the equatorial line. (**c**) Schematic of V-effusion cell, VTIP gas injector and 1 × 1 cm substrates arrangement for the growth of VO_2_ films with varying equivalent VTIP fluxes from sample to sample. (**d**) Resistivity at 30 (insulating state) and 80 °C (metallic state) of five 1 × 1 cm samples grown using the set-up in **c**. (**e**) Calculated equivalent VTIP flux distribution across a 3-inch wafer with 4.5 × 10^−7^ torr VTIP ion gauge pressure. For size comparison, the green square at the center represents a 1 × 1-cm sample. The colour key provided in **e** also applies to **b**,**d** and **f**. **(f)** Superposition of the resistivity data shown in **b** and **d**. The position axis in **b** was converted to equivalent VTIP flux using the flux profile calculated from equation (1) that is shown in **e**.

Figure 1a shows the schematic set-up in the growth chamber. A V-metal source and a VTIP source were mounted on diametral flanges, aligning the flux gradient of V and VTIP in opposite direction to increase their flux ratio gradient. The flux density at position *p* on the sample is given by:^39^


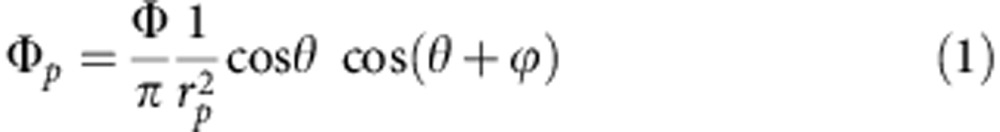


with Φ the flux density effusing from the source, *r*_*p*_ the distance between the centre of the crucible orifice and the point *p* on the substrate. Angle *θ* and *ϕ* are the effusing angle and the angle of effusion cell with respect to the substrate normal at the centre. V and VTIP sources each generated a flux gradient across the substrate. The flux variation for both sources was calculated as +18 and –16% at the sample positions 3.8 cm off centred towards and away from the source on the equatorial line. Therefore, the total change of the flux ratio 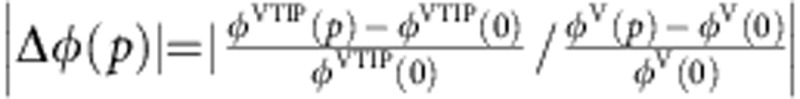 ranged from 140 to 71%. For simplicity, an equivalent VTIP flux 
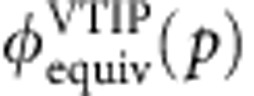
 was defined at position *p* on the wafer to represent the VTIP:V flux ratio. At the centre of the substrate, 
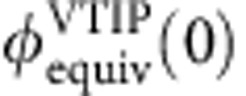
 was set to be equal to the VTIP ion gauge pressure measured at the centre of sample position before growth, while the equivalent VTIP flux at position *p* was defined as 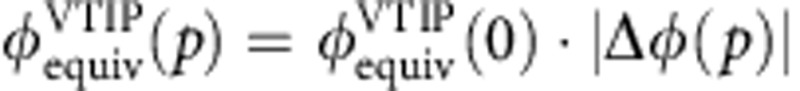.

To create a VO_2_ valence state library on a 3-inch *r* plane sapphire substrate, the wafer was not rotated during growth. For the supply of VTIP, the flux was set to an beam equivalent pressure of 4.5 × 10^−7^ torr determined by a beam flux ion gauge at the sample position, while V-flux was fixed as 4 × 10^12^ cm^−2^ s^−1^, measured by a quartz crystal microbalance. Resistivity measurements at two different temperatures, 30 (insulating state) and 80 °C (metallic state) were taken on devices every 7.5 mm along the equatorial line of the wafer and are plotted in Fig. 1b. For the position on the wafer closest to the V effusion cell (−3 cm), a small resistivity ratio Δ*ρ*=(*ρ*_30 °C_−*ρ*_80 °C_)/*ρ*_80 °C_ of only 4.7 was measured. The resistivity ratio progressively increased towards the centre of the wafer. While the resistivity of the metallic state at 80 °C remained almost constant, the resistivity of the insulating phase at 30 °C increased exponentially. At wafer positions closer to the V-cell, the low resistivity of the insulating phase was attributed to a pronounced oxygen deficiency in the film, which increased the unintentional carrier concentration via oxygen vacancy formation^21^,^30^. A similar trend in the Δ*ρ* was also observed for wafer positions closer to the VTIP gas injector (3 cm). In contrast, here the resistivity of the metallic state at 80 °C sharply increased with distance away from the wafer centre, attributed to enhanced scattering from secondary oxygen-rich phases^8^. The highest quality film, indicated by the largest resistivity ratio Δ*ρ*=1.1. × 10^4^ was found at *x*=0 cm. X-ray photoelectron spectroscopy (XPS) measurements performed along the equatorial line confirmed the existence of an oxygen activity gradient during growth and its direct effect on the valence state of vanadium in the film (details in the Supplementary Note 1 and Supplementary Fig. 1).

To further confirm the validity of the combinatorial growth approach and the calculated flux gradient, five 30-nm-thick VO_2_ films were grown on 1 × 1 cm *r* plane sapphire substrate mounted at the centre of the substrate holder and were rotated at 3 r.p.m. to ensure good uniformity. Throughout this series, the V-flux was kept as 4 × 10^12^ cm^−2^ s^−1^ while the VTIP flux, determined as beam equivalent pressure at the sample position, was varied from 2.5 × 10^−7^ to 6.5 × 10^−7^ torr, see Fig. 1c. A similar resistivity trend as function of equivalent VTIP fluxes was reproduced and is shown in Fig. 1d. The resistivity results from both experiments (Fig. 1b,d) are shown in Fig. 1f for direct comparison. Using the calculated flux gradient in Fig. 1e, the positions along the equatorial line in Fig. 1b were converted into equivalent VTIP fluxes. The trends in resistivity with changes in the equivalent VTIP flux are well matched, evidencing the feasibility of the combinatorial approach to optimize cation-to-anion ratios in VO_2_ films.

### Structural and physical properties of 3-inch VO_2_ films

**Figure 2:**
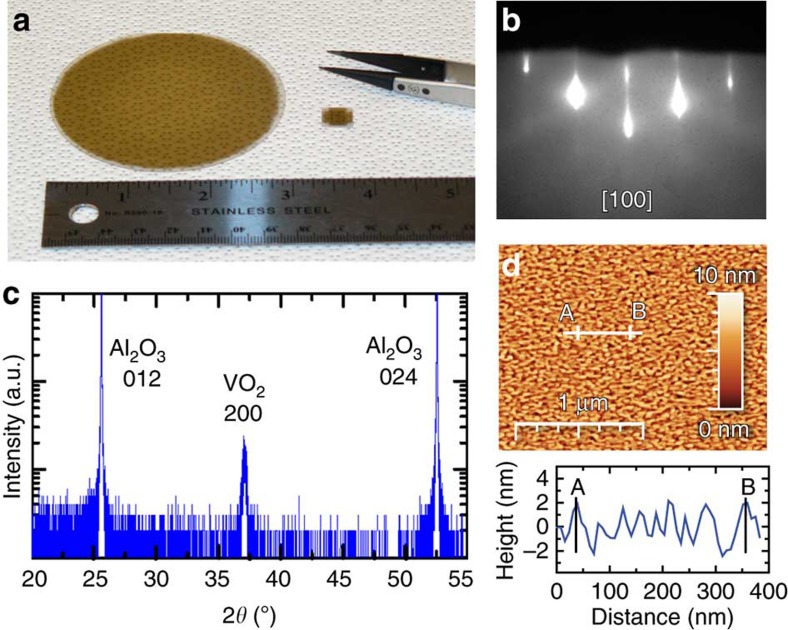
Characterization of VO_2_ films grown on a 3-inch *r* plane sapphire substrate. (**a**) Comparison of substrate sizes used for the VO_2_ thin-film growth. (**b**) Streaky RHEED pattern of VO_2_ films observed along [100] azimuth after growth. (**c**) Wide range 2*θ*−*ω* X-ray diffraction scans of VO_2_ grown on a *r* planeAl_2_O_3_ substrate. (**d**) AFM scan of the film surface morphology and a line scan along AB.

The approach to co-supply V and VTIP was used to demonstrate that a scale up of highly uniform VO_2_ films can be achieved when the substrate is rotated. A 30-nm-thick film was deposited on a 3-inch sapphire at 350 °C and rotated at 3 r.p.m., while supplying a VTIP flux with beam equivalent pressure of 4.5 × 10^−7^ torr, and a V-flux of 4 × 10^12^ cm^−2^ s^−1^. Figure 2a shows a direct comparison of the VO_2_ film grown on a 3-inch wafer to the film grown on a 1 × 1-cm substrate, both exhibiting good uniformity. Reflection high-energy electron diffraction pattern (RHEED) taken during and after the growth remained streaky, indicating an epitaxial film with very good surface roughness and crystallinity, which did not change when scanning the RHEED beam spot across the wafer, see Fig. 2b. X-ray diffraction 2*θ*−*ω* scans taken at the wafer centre revealed the absence of any other vanadium oxide phase but VO_2_ with an out-of-plane orientation VO_2_ (100)||Al_2_O_3_ (012) (refs 40, 41), see Fig. 2c. In plane X-ray diffraction Φ scans of the VO_2_ 220 peak were carried out with 2*θ*=55.45° and *ψ*=46.86°, demonstrating a good in plane epitaxy with twofold symmetry, similar to previous report of epitaxial VO_2_ film on *r* plane sapphire^22^, see Supplementary Fig. 2. The film surface was found to be smooth, yet not atomically flat, with a root mean square roughness of 1.1 nm. The surface morphology shown in Fig. 2d was dominated by small irregularly shaped islands separated by corrugations of typically 1–2 nm, commonly observed for VO_2_ films grown on Al_2_O_3_ (ref. 21).

**Figure 3:**
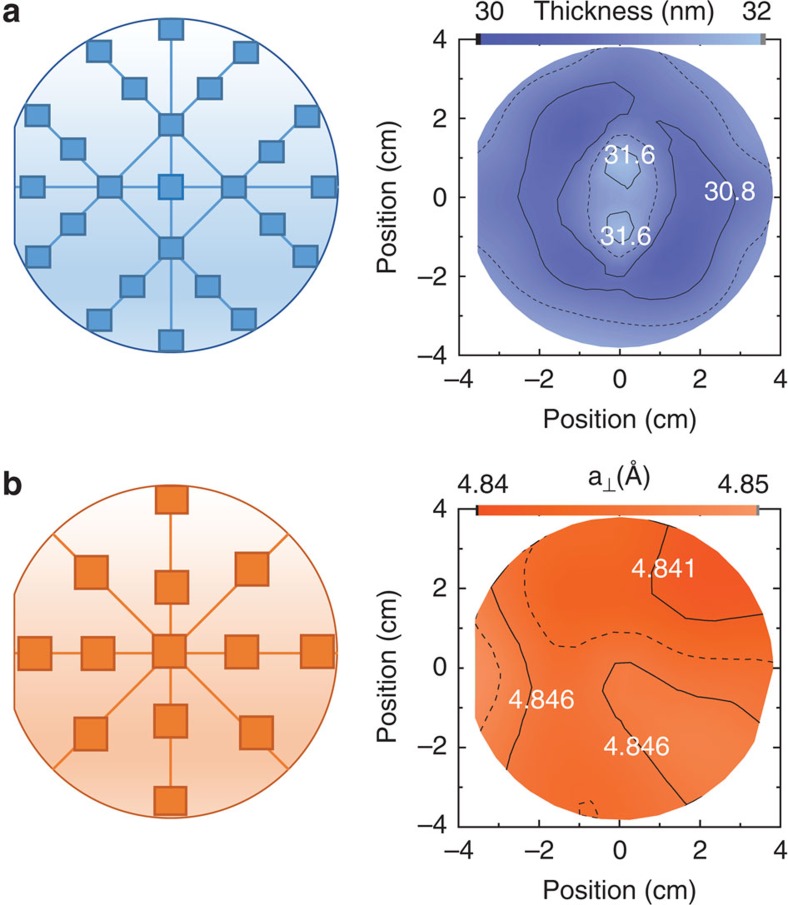
Wafer-scale metrology of VO_2_ grown on a 3-inch *r* plane sapphire substrate. The squares in the schematic on the left represent the probing locations on the wafer. (**a**) Film thickness uniformity extracted from spectroscopic ellipsometry measurements. (**b**) Map of the out-of-plane lattice parameter determined from X-ray diffraction scans.

**Figure 4:**
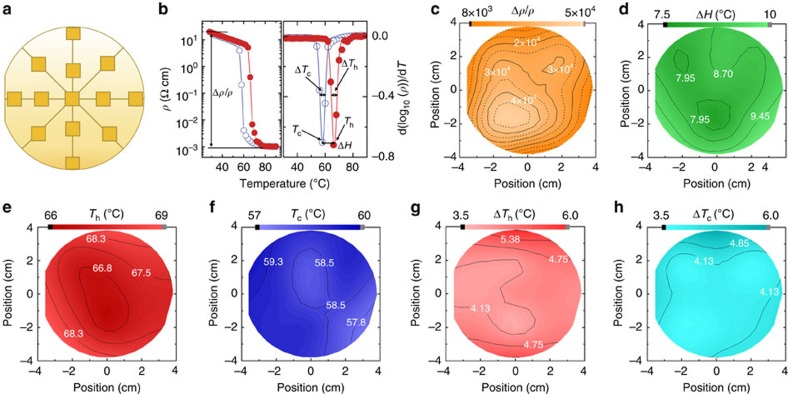
Wafer-scale mapping of MIT properties of 3-inch VO_2_. (**a**) Schematic with squares showing the probing locations on the wafer. (**b**) Definition of the MIT properties: resistivity ratio (Δ*ρ*/*ρ*), transition point (*T*_h_ and *T*_c_), transition sharpness (Δ*T*_h_ and Δ*T*_c_) and transition width (Δ*H*). The subscripts h and c indicate the cooling and heating cycle, respectively. Closed red and open blue circles represent heating and cooling cycles, respectively. (**c**) Map of VO_2_ resistivity ratio Δ*ρ*/*ρ*=(*ρ*_30 °C_−*ρ*_80 °C_)/*ρ*_80 °C_ obtained from four-point–probe measurements of resistivity *ρ*_30 °C_ at 30 °C (VO_2_ insulating state) and *ρ*_80 °C_ at 80 °C (VO_2_ metallic state). Wafer-scale mapping of MIT properties defined in **b** across the 3-inch VO_2_ film: (**d**) Δ*H*, (**e**) *T*_h_, (**f**) *T*_c_, (**g**) Δ*T*_h_, (**h**) Δ*T*_c_.

Figure 3 shows the wafer-scale metrology of the 3-inch VO_2_ thin film, where the film was characterized at multiple positions across the sample using spectroscopic ellipsometry (SE) and X-ray diffraction measurements. The film thickness profile as a function of position was extracted from SE measurement and is shown in Fig. 3a. Overall, the film thickness was uniform, with a s.d. of 0.37 nm (1.2%), which is well within the technical specifications of the MBE system. The thickness was maximum at the wafer centre, and continuously decreased towards the wafer edges. X-ray diffraction was used to map out the VO_2_ film out-of-plane lattice parameter, as shown in Fig. 3b. In contrast to the thickness variation, the lattice parameter remained virtually unaffected with a s.d. of 0.002 Å, limited by the precision of the X-ray diffraction measurement. Good uniformity of the MIT properties (resistivity ratio, transition point, sharpness and width) was confirmed by testing devices across the wafer. Figure 4a shows the locations and Fig. 4b the definitions of the MIT properties analysed. The resistivity ratio across the MIT is shown in Fig. 4c. More than 71% of the wafer area had resistivity ratio Δ*ρ* >2 × 10^4^, a remarkable combination of high yield, uniformity and quality of wafer-scale VO_2_ films. In addition to the resistivity ratio, other MIT properties were also characterized across the 3-inch film, see Fig. 4 d–h. The derivative of log_10_ (*ρ*) as a function of temperature was plotted and fitted with a Gaussian function. The transition points of the heating and cooling cycle, *T*_h_ and *T*_c_, were defined at the respective peak position of the Gaussian and the transition width Δ*H*=*T*_h_−*T*_c_ was directly calculated. The transition sharpness Δ*T*_h_ and Δ*T*_c_ were determined from the full-width at half-maximum of the Gaussian peak. The properties were proved uniform, with an s.d. of 0.64 and 0.47 °C for *T*_h_ and *T*_c_, 0.35 and 0.41 °C for Δ*T*_h_ and Δ*T*_c_, and 0.61 °C for Δ*H*.

**Figure 5:**
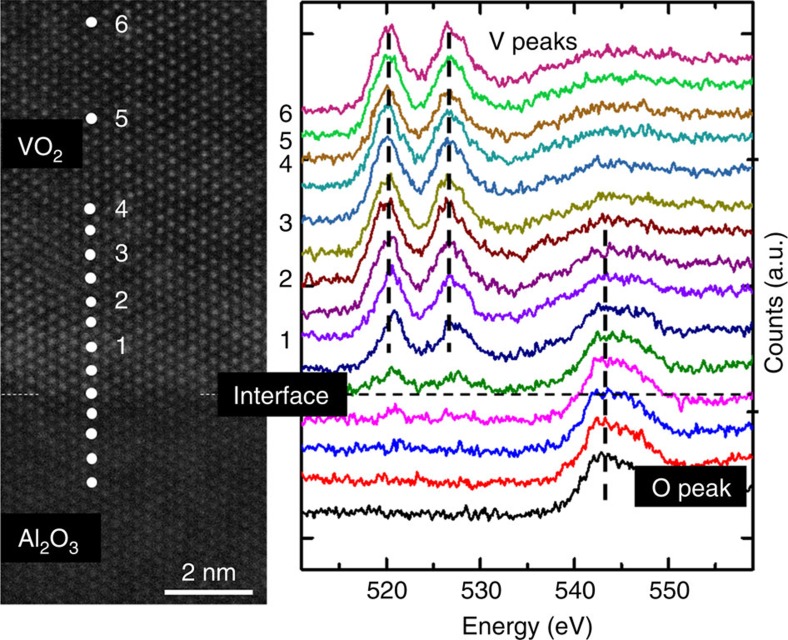
STEM and EELS characterization. High-angle annular dark-field scanning transmission electron microscopy (HAADF-STEM) image of the VO_2_/Al_2_O_3_ interface. Since Vanadium has a higher atomic number compared with Aluminum (23 versus 13), the film can be distinguished compared with the substrate by the higher intensity vanadium atoms. Electron energy loss spectroscopy (EELS) to determine the vanadium valence across the interface is shown on the right. EELS spectra were taken at the position indicated in the STEM image. The EELS data were collected from a beam spot size with ∼1-Å diameter.

To expand the characterization of the film quality to other film properties and to provide film characterization across the wafer but with mesoscale resolution, Kelvin probe force microscopy (KPFM) measurements were performed at various positions, to extract the VO_2_ work function at room temperature as well as its uniformity across the 3-inch wafer. The work function was found to be uniform across the wafer with an average value of 4.85±0.10 eV, agrees with previous reports of VO_2_ work function measured by KPFM^25^,^42^ and ultraviolet photoelectron spectroscopy^43^ (details in the Supplementary Note 2 and Supplementary Fig. 3). To characterize the film structural quality and the vanadium valence state of the VO_2_ film at the atomic scale, high-angle annular dark-field scanning transmission electron microscopy and electron energy loss spectroscopy (EELS) measurements were performed across the VO_2_/Al_2_O_3_ interface. Figure 5 shows an annular dark-field scanning transmission electron microscopy image of an epitaxial sharp interface between VO_2_ and Al_2_O_3_. Line scan EELS spectra were taken with 0.5 nm step size starting from the substrate into the film to probe the evolution of the valence state of vanadium. The low-energy loss peak determined for the film was around 520 eV, which did not shift with scan position and remained between 519 and 521.5 eV measured for the V_2_O_3_ and V_2_O_5_ calibration samples, respectively (details in the Supplementary Fig. 4). The shift of EELS peaks between V_2_O_3_, VO_2_ and V_2_O_5_ observed here agrees with the trend previously reported^44^, showing dominant valence state of V to be 4^+^ state in the film and at the vicinity of the interface. In particular, the absence of a wider transition layer, which was previously observed for VO_2_ films grown on *c* plane sapphire and attributed to the formation of oxygen-deficient Magnéli phases V_*n*_O_2*n*−1_ (ref. 45), demonstrates the excellent control over the V-oxidation state right from the film growth initialization.

**Figure 6:**
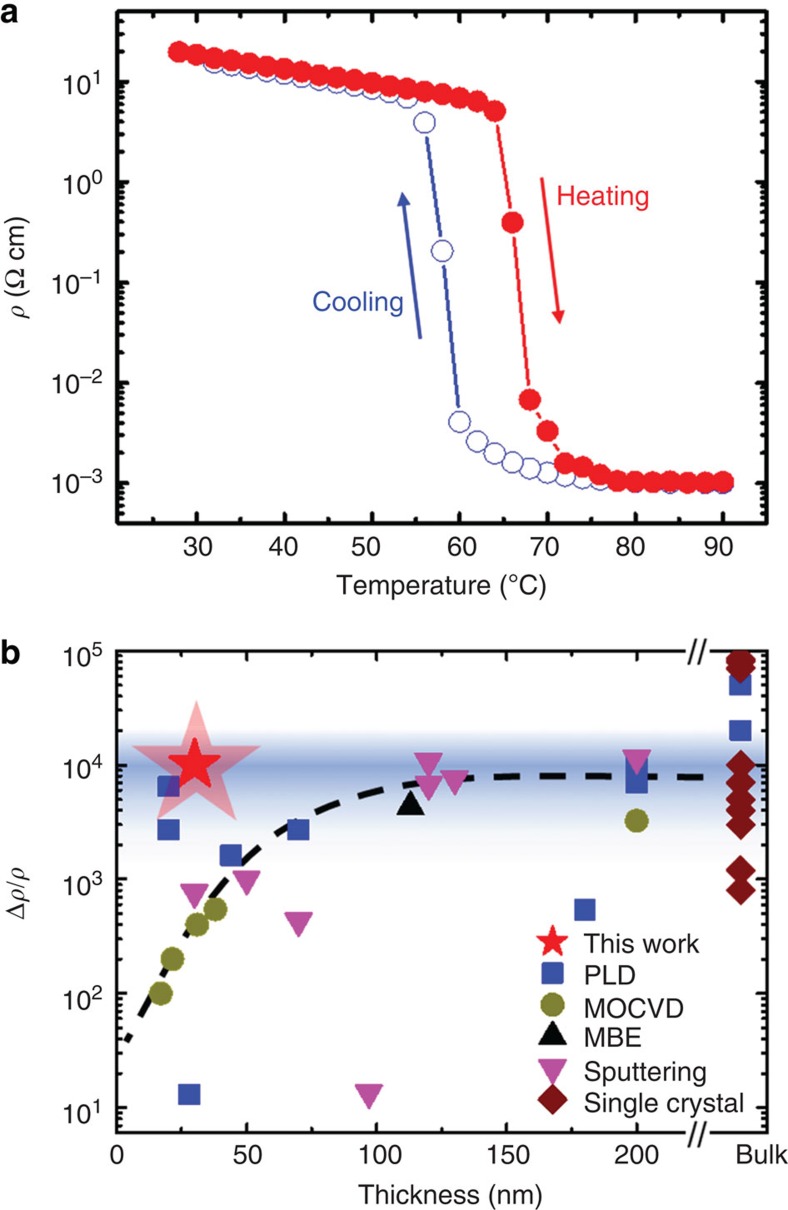
Benchmark of VO_2_ thin films. (**a**) Temperature-dependent resistivity of VO_2_ films grown under optimized condition on a 3-inch *r* plane Al_2_O_3_. Measurement was taken at the wafer center. (**b**) Benchmark of resistivity ratios Δ*ρ*/*ρ*=(*ρ*_50 °C_−*ρ*_80 °C_)/*ρ*_80 °C_ for VO_2_ across the metal-to-insulator transition. Note that the low temperature resistivity was taken at 50 °C. For a direct comparison, all films were grown on Al_2_O_3_ substrates above the critical films thickness. The blue-shaded area indicates the highest values reported for VO_2_ films on sapphire and typical values obtained in bulk single crystals. A dotted line was added as guide to the eye. Note the two PLD samples with resistivity ratios higher than Δ*ρ*/*ρ*=10^3^ at film thicknesses <25 nm, from ref. 21. A thickness much >200 nm was estimated for the VO_2_ films grown by PLD in ref. 57 and the data were added as ‘bulk' value. Data for bulk VO_2_ were taken from refs 47, 48, 49, 50, 51, 52, 53. Properties of VO_2_ films on Al_2_O_3_ substrate grown by PLD were taken from refs 21, 42, 52, 53, 54, 55, 56, 57, MOCVD from refs 58, 59, MBE from ref. 27 and sputtering from refs 8, 23, 25, 60, 61, 62, 63.

To benchmark the quality of VO_2_ thin film, temperature-dependent resistivity has been measured for a device at the wafer center and is shown in Fig. 6a. A representative set of data was compiled and is shown in Fig. 6b to allow for a direct comparison with bulk single crystals^46^,^47^,^48^,^49^,^50^,^51^,^52^ as well as VO_2_ thin films grown by deposition techniques such as PLD^21^,^41^,^53^,^54^,^55^,^56^, metalorganic chemical vapour deposition^57^,^58^, MBE^27^ and sputtering^8^,^23^,^25^,^59^,^60^,^61^,^62^. The selection criteria were either single crystals in case of bulk material, or completely relaxed VO_2_ films grown on sapphire samples, that is, with thicknesses exceeding the critical film thickness of 20 nm (ref. 21). Here the change of resistivity Δ*ρ*/*ρ* across the MIT was defined as Δ*ρ*/*ρ*=(*ρ*_50 °C_−*ρ*_80 °C_)/*ρ*_80 °C_≈*ρ*_50 °C_/*ρ*_80 °C_, which serves as a benchmark to compare material quality. For the best bulk single crystal, a resistivity ratio of Δ*ρ*/*ρ*=9 × 10^4^ was reported^46^,^47^, while the majority of resistivity ratios achieved in bulk single crystals were much lower than that with an average of ∼5 × 10^3^ and not exceeding 1 × 10^4^ (refs 47, 48, 49, 51, 52). Resistivity ratios for VO_2_ thin films varied significantly with the highest values Δ*ρ*/*ρ* being comparable to the majority of those reported for bulk single crystals (shaded area in Fig. 6b). Notably, a reduction in Δ*ρ*/*ρ* was found to occur with decreasing film thickness, likely due to the formation of an interfacial transition layer^45^, which can occur if the cation-to-anion stoichiometry was not maintained throughout the entire growth. If this nonstoichiometric layer amounts to a significant fraction of the film, the resistivity ratio is ultimately compromised. However, the 30-nm-thick VO_2_ film grown here only showed an ultrathin transition layer at the vicinity of the VO_2_/Al_2_O_3_ interface (Fig. 5) and revealed a four orders of magnitude change in resistivity across the MIT, similar to the largest value reported by Jeong *et al.*^21^ for ultrathin VO_2_ films grown by PLD on sapphire and on par with the majority of bulk single-crystal values^47^,^48^,^49^,^51^,^52^. To demonstrate the reproducibility of this technique and its versatile applicability to challenging templates, similar optimization processes were repeated on a 3-inch *c* plane sapphire wafer and on the technologically interesting, but more challenging silicon substrate. Details are given in the Supplementary Notes 3 and 4 and Supplementary Figs 5 and 6. While a similarly high-resistivity ratio with four orders of magnitude change was obtained on *c* plane sapphire along the equatorial line of the wafer, the resistivity ratio of VO_2_ on silicon was reduced, which was attributed to the polycrystallinity of the film induced by the amorphous substrate that resulted in a high surface roughness and high grain boundary density^63^.

## Discussion

It is demonstrated that the highly desirable combination of superior material quality in VO_2_ thin films, with resistivity ratios rivalling the highest values reported for thin films and typical values obtained for bulk VO_2_ single crystals, excellent uniformity across a 3-inch wafer, and high yield is possible. The precise valence state control was achieved by a highly efficient combinatorial growth strategy, where a continuous vanadium valence state library was established across the wafer by supplying oxygen in a pre-bonded form. The ability to grow VO_2_ thin films with excellent reproducibility and minimal calibration time on wafer scale overcomes a critical roadblock, making it feasible to transfer VO_2_-based functionalities into viable technologies and directly impacting different application spaces, such as thermochromics^64^ or memory metamaterials^18^. This approach is not limited to VO_2_, but can be further extended to other oxide systems containing multivalent cations, therefore providing a new gateway to grow large scale multivalent oxides with superior valence state control.

## Methods

### Combinatorial growth of VO_2_ thin films

Growth was carried out in DCA M600 MBE growth chamber with a base pressure of 5 × 10^−10^ torr. VO_2_ thin films were grown on a 3-inch (Precision Micro-Optics LLC) and 1 × 1-cm (MTI Corporation) *r* plane sapphire substrates via co-supply of VTIP (vacuum distilled, trace metal impurity 4N, MULTIVALENT Laboratory) and vanadium metal (4N, Ames Laboratory). VTIP was kept in a bubbler (heated to 50 °C), connected to the MBE growth chamber through a heated gas inlet. A low-conductance gas injector was used to supply VTIP to the substrate. A proportional-integral-derivative (PID)-controlled adjustable leak valve and capacitance manometer were used to maintain a constant VTIP gas inlet pressure. An ion gauge was used to monitor VTIP flux, which was positioned at the center of the sample position before growth. Vanadium metal was evaporated from a high temperature effusion cell. V-flux calibration was performed using a quartz crystal microbalance at growth position. Substrates were cleaned in ultrasonic bath of acetone and then isopropanol before loading them into the MBE system and baked at 150 °C for 2 h in the load lock chamber. The substrates were transferred into the MBE growth chamber and heated to the film growth temperature of 350 °C. Before deposition substrates were exposed for 20 min in oxygen plasma (250 W, 9 × 10^−7^ torr background pressure). The growth was carried out by co-supplying V (4 × 10^12^ cm^−2^ s^−1^) and VTIP in the presence of an oxygen plasma. Film growth rate was 5 Å min^−1^. After growth the films were cooled in the presence of oxygen plasma.

### VO_2_ thin-film characterization

Film growth was monitored *in situ* using RHEED. XPS measurement was performed on PHI VersaProbe with 200 μm spot size. Aluminum Kα radiation was used with a pass energy of 29.32 eV. Fitting was performed using CasaXPS software (Supplementary Note 1). Surface morphology of the VO_2_ thin film was characterized using a Bruker Dimension Icon Atomic Force Microscope operated in peak force tapping mode. KPFM measurements were carried out with the same set-up as atomic force microscope using conductive Pt-coated Si cantilevers. To reduce the effect of surface water absorption, samples were measured immediately after growth. Highly ordered pyrolytic graphite was used as a reference sample to calibrate the cantilever potential. During the measurement, an a.c. voltage with the amplitude of 3 V and frequency of 60 kHz was applied to the cantilever. X-ray diffraction was carried out by a Phillips X'Pert Panalytical MRD Pro thin-film diffractometer equipped with a duMond–Hart–Partels Ge (440) incident beam monochromator using Cu Kα_1_ radiation. The uniformity of VO_2_ film thickness was characterized *ex situ* using room temperature SE (M-2000U, J. A. Woollam) in the spectral range from 1.125 to 5.175 eV. Data were fitted using a model comprised of two layers: VO_2_ thin film and *r* plane sapphire substrate. The sapphire substrate was approximated using a Cauchy model


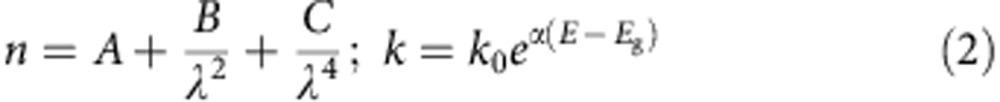


with *E*_g_=8.8 eV. The VO_2_ thin films were modelled using three Tauc–Lorentz oscillators


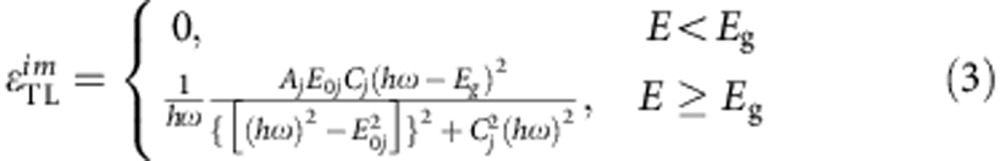


with parameters 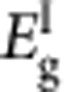
=0.541 eV, 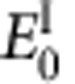
=1.144 eV, 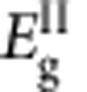
=1.857 eV, 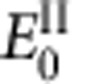
=3.469 eV, 
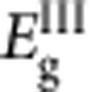
=3.568 eV and 
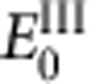
=4.807 eV, corresponding to optical interband transitions: 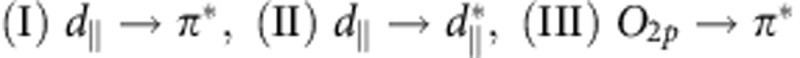 (refs 65, 66). Very good agreement between model and experiment was obtained with a root mean square error of ∼2. Temperature-dependent resistivity measurements were performed in the van der Pauw geometry on a Cascade probe station, where 5 × 5 mm pieces were cut from the wafer for resistivity measurement. Contour figures were plotted using Origin software with triangulation and linear interpolation method between the data points. High-angle annular dark-field scanning transmission electron microscopy images and EELS spectra were collected using the FEI Titan^3^ 80–300 microscope operating at 200 kV in the STEM mode. The EELS spectra were collected using dual 2048 channel Gatan CCD detectors at a semi-angle of 21.4 mrad with an energy dispersion of 0.1 eV per channel. The L_2,3_ edge peak position in the vanadium films were compared with V_2_O_3_ and V_2_O_5_ powder reference samples, obtained from Sigma-Aldrich.

## Additional information

**How to cite this article:** Zhang, H.-T. *et al.* Wafer-scale growth of VO_2_ thin films using a combinatorial approach. *Nat. Commun.* 6:8475 doi: 10.1038/ncomms9475 (2015).

## Supplementary Material

Supplementary Information Supplementary Figures 1-6, Supplementary Notes 1-4 and Supplementary References
