# Supplementary material for: Wafer-scale growth of VO2 thin films using a combinatorial approach
Source: Nat Commun. 2015 Oct 9;6:8475. doi: 10.1038/ncomms9475 (PMC4633718; doi:10.1038/ncomms9475)
Supplement: Supplementary Information — Supplementary Figures 1-6, Supplementary Notes 1-4 and Supplementary References [file ncomms9475-s1.pdf]

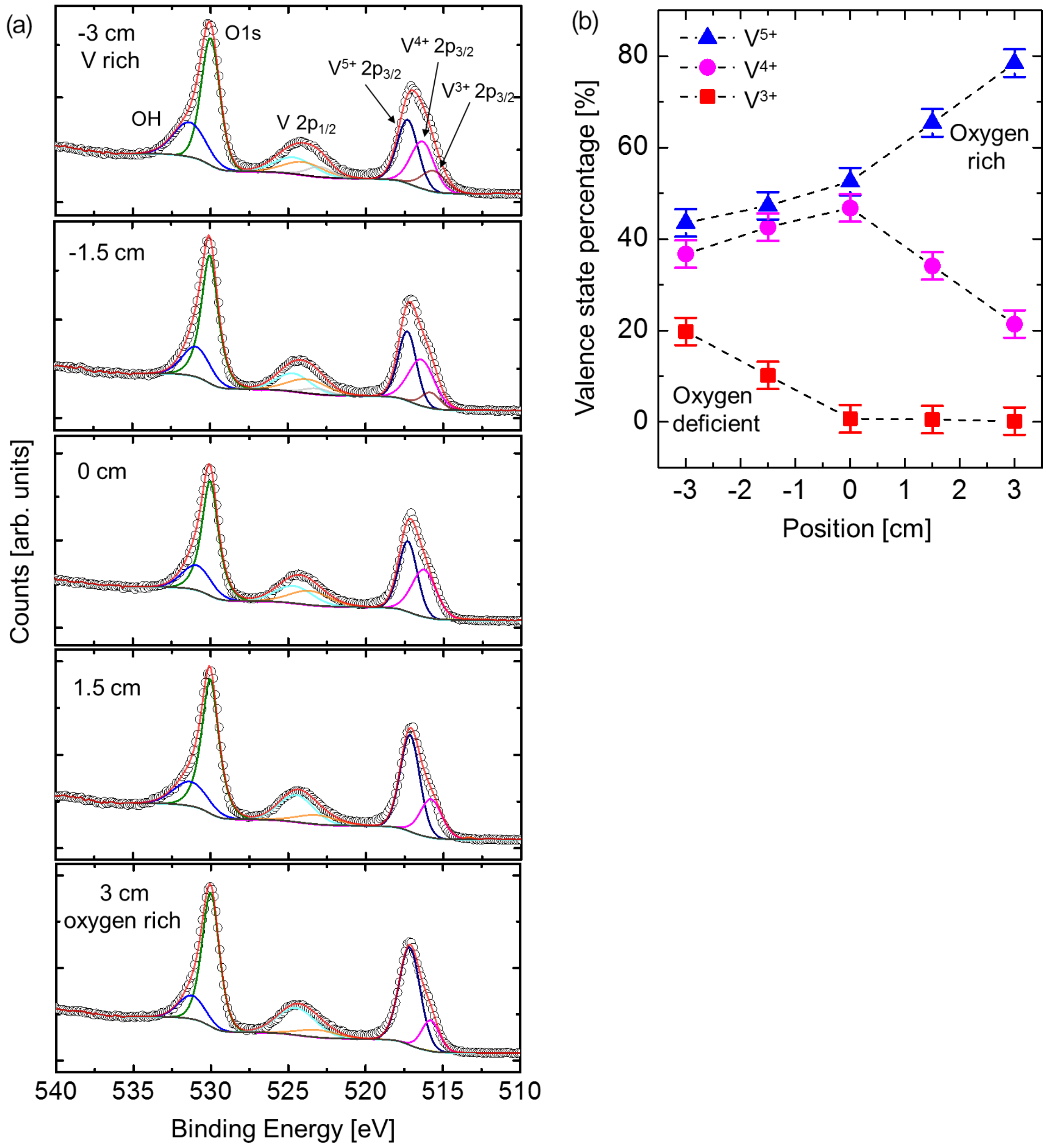

Supplementary Figure 1. X-ray photoelectron spectroscopy (XPS) measurements. (a) The XPS scans around V2p peak and corresponding fitting result. The dotted line corresponds to the measured data and the red line is the envelope of the modeled peaks. **(b)** Relative area of the of V^{3+}, V^{4+} and V^{5+} XPS peaks as a function of position along the oxygen activity gradient. Error bars give a conservative estimate of ±2%, outside of which a good match of calculated and measured XPS scans cannot be achieved.

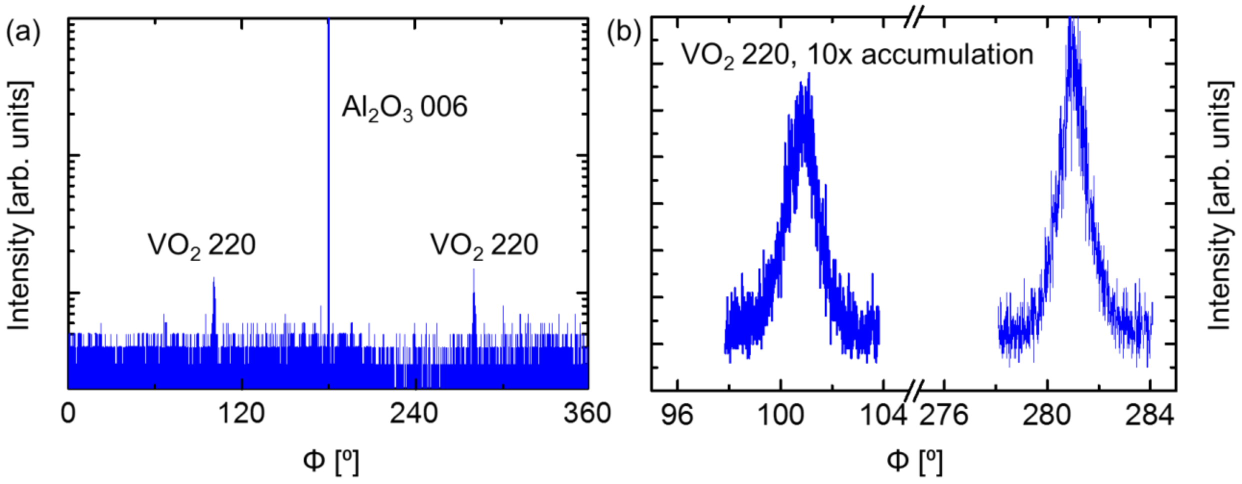

Supplementary Figure 2. XRD Φ scan of VO_2 grown on r–sapphire. (a) XRD in plane wide angle Φ scan of the VO_2 220 film peak and the Al_2O_3 006 substrate peak. **(b)** Small angle Φ scan of the VO_2 220 peak with 10 times longer accumulation time.

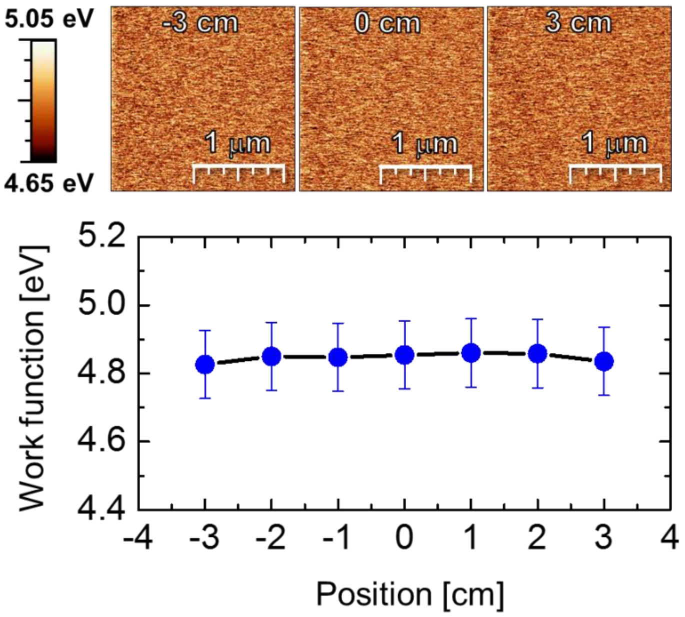

Supplementary Figure 3. **Kelvin probe force microscopy measurements of VO_2 grown on r-sapphire.** The upper panel shows the spatial maps of the work function taken at the wafer center (0 cm) and edges (±3 cm). The lower panel shows the work function averaged from the work function spatial maps taken at different positions across the wafer. Error bars are estimated to be the ±0.1 eV, which correspond to the full width at half maximum (FWHM) of the measured potential histogram.

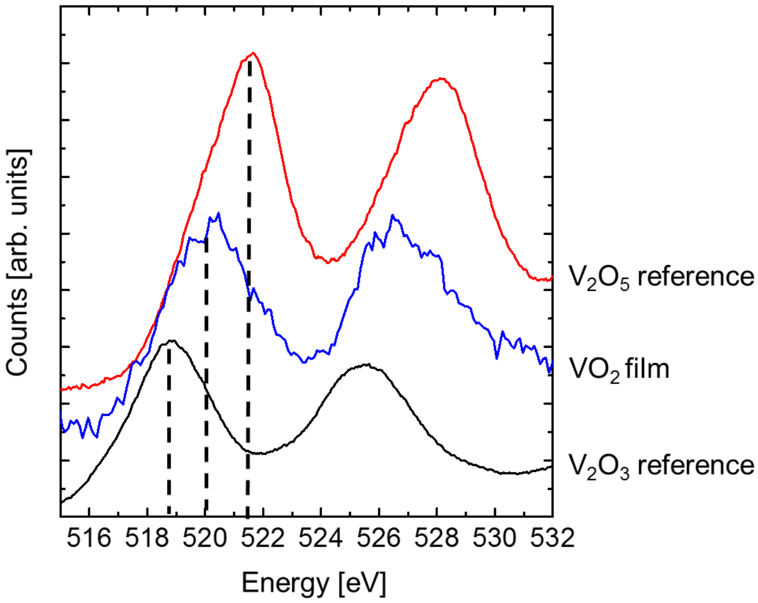

Supplementary Figure 4. **EELS reference spectra.** EELS spectra at vanadium L edge for standard calibration samples and the VO_2 film.

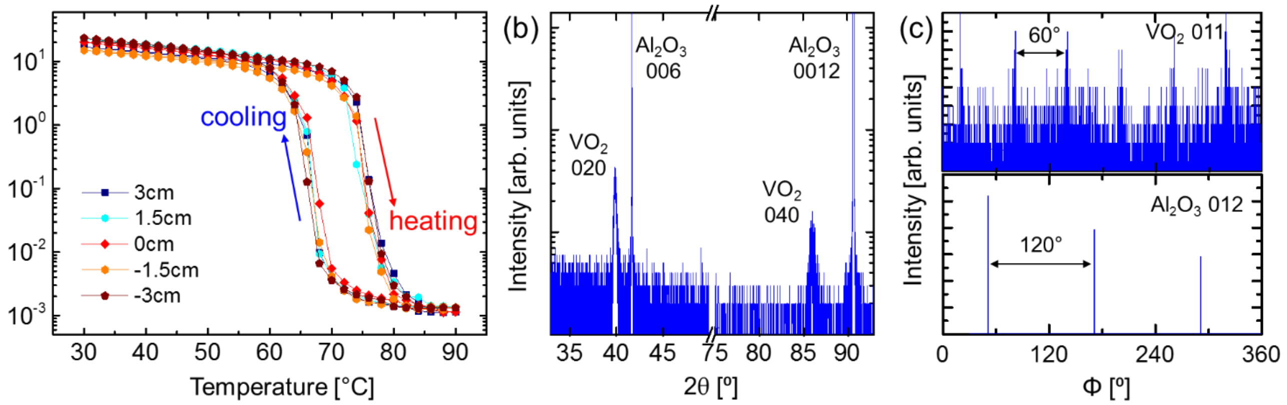

Supplementary Figure 5. Wafer scale VO_2 films grown on c-sapphire. (a) Temperature dependent resistivity measurements of a 30-nm VO_2 film grown at optimized condition on c-Al_2O_3, taken at five locations along the diameter of the 3 inch wafer. **(b)** Wide range 2θ-ω X-ray diffraction (XRD) scans of VO_2 grown on c-Al_2O_3 substrate. **(c)** In plane XRD Φ scans of the VO_2 011 film peak and the Al_2O_3 012 substrate peak.

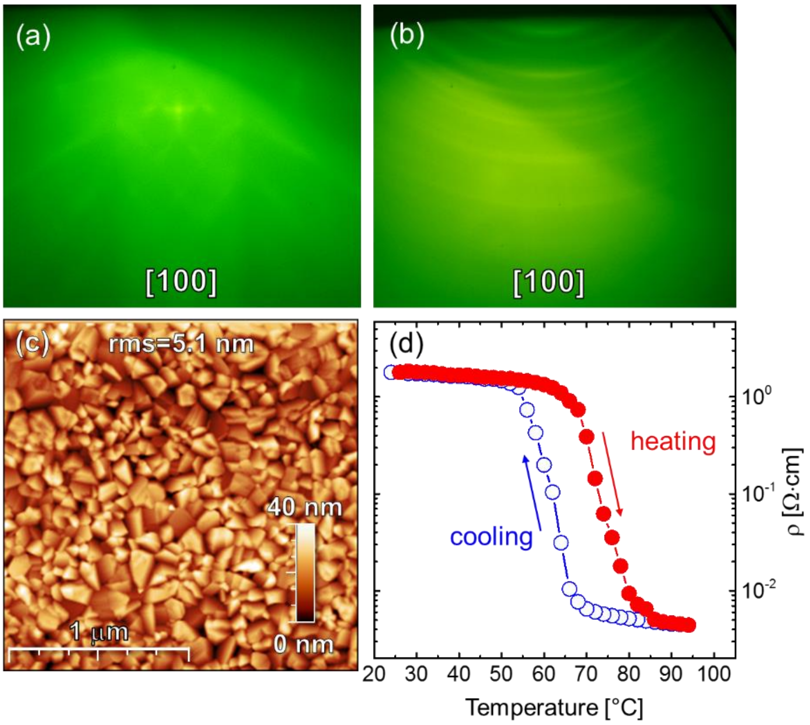

Supplementary Figure 6. Wafer scale VO_2 films grown on silicon. (a) Reflection high energy electron diffraction (RHEED) pattern of the Si (001) substrate along [100] azimuth before deposition, indicating an amorphous native oxide layer on top of Si. **(b)** RHEED pattern of 30-nm-thick VO_2 grown on Si substrate under optimized condition. Ring shaped pattern indicated polycrystalline VO_2. **(c)** AFM scan of the film surface, showing polycrystalline microstructure with a rough surface morphology (rms value 5.1nm). **(d)** Temperature dependent resistivity of VO_2 film grown under optimized condition on Si.

Supplementary Note 1 X-ray photoelectron spectroscopy (XPS) measurement along the path of resistivity change across the 3 inch film

Five spots along the equatorial line (1.5 cm separation) were measured by XPS to directly demonstrate the changes of the vanadium valence state in the film and to confirm the existence of an oxygen activity gradient during growth Supplementary Figure 1(a) shows high resolution XPS scans of V 2p and O 1s core levels and the corresponding fits to the spectra. CasaXPS software was used for Shirley background subtraction and to perform a Marquardt algorithm-based fitting using the routine and peak parameters based on the recommendation given by Silversmit *et al.*[1] for the quantitative determination of V valence states. The O_{1s} peak was used as reference at a fixed peak position of 530 eV (oxygen in VO_x). $V5^+$ $2p_{3/2}$ peak position was restricted to (517.5±0.2) eV, $V4^+$ $2p_{3/2}$ peak to (516.0±0.2) eV and $V3^+$ $2p_{3/2}$ peak to (515.5±0.2) eV. Additional fitting parameter constraints were used: 1). the energy split between the V $2p_{3/2}$ and V $2p_{1/2}$ peak was fixed to 7.33 eV. 2). the peak area of V $2p_{3/2}$ was twice that of V $2p_{1/2}$ peak. An additional O1s peak was defined at a higher binding energy than 530 eV and assigned to OH. Good match between the model and the experimental spectra were obtained.

Supplementary Figure 1(b) shows the percentage of each valence state calculated from the integrated area of corresponding XPS peak. While at the Ox-rich side (low conductivity at metallic state) the V^{5+} was the dominant valence state, smallest amount of V^{5+} and the largest amount of V^{3+} was found on the Ox-deficient side (low resistivity at insulating state). Absence of V^{3+} and the highest intensity for V^{4+} was found in the center of the wafer. The ubiquitous presence of the V^{5+} component was due to the oxidation of the vanadium oxide surface in ambient. Note that the surface oxidation effect was relatively small, the 'overlayer' thickness was estimated be to around 0.8 nm. The overlayer was estimated from the dataset measured at the wafer center position, assuming that the V^{5+} signal came only from the overlayer with an inelastic mean free path of 1.6 nm. These measurements confirm the existence of an oxygen activity gradient across the wafer during growth and its direct effect on the valence state of vanadium in the film.

Supplementary Note 2 Kelvin probe force microscopy (KPFM) measurement of the VO_2 work function

While the 3 inch film showed uniform MIT properties, physical properties were also proved uniform. Kelvin probe force microscopy (KPFM) were used to measure the VO_2 work function at seven 2×2 μm^2 spots along the diameter of freshly grown VO_2 film on 3 inch sapphire, see Supplementary Figure 3. The upper panel showed the spatial maps of the work function at the sample center (0 cm) and edges (±3 cm). Despite the granular surface morphology of the VO_2 film seen in AFM measurement [Fig. 2(d)], the work function of the film remains homogeneous in the spatial map, indicating a uniform stoichiometry. Along the sample diameter, the work function of the VO_2 film remains uniform (Supplementary Figure 3 lower panel), with an averaged value of 4.85 eV, agrees with previous reports of VO_2 work function measured by KPFM[2,3] and ultraviolet photoelectron spectroscopy (UPS).[4]

Supplementary Note 3 Growth of VO_2 on c-plane sapphire

Supplementary Figure 5(a) shows the temperature dependent resistivity measurements of five spots along the diameter of a 30-nm VO_2 film grown under optimized condition on a 3 inch c-plane sapphire wafer. Similar to the film grown on r-plane sapphire, four orders of magnitude change in resistivity was observed across the MIT, with uniform transition characteristics across the 3 inch film (transition temperature, sharpness, etc.). The epitaxial relationship between film and c-plane sapphire substrate was determined by XRD 2θ-ω and Φ scans. In Supplementary Figure 5(b), the 2θ-ω scan showed, except for substrate peaks, two VO_2 peaks at 2θ=39.89° and 2θ=85.96°. The peak at smaller 2θ can be attributed to monoclinic VO_2 020 or 002, the peak at higher 2θ to monoclinic VO_2 040 or 004, respectively.[5] In plane Φ scans were performed for the off-axis monoclinic VO_2 peaks 011 (2θ=27.88°, ψ=44.93°) and $\bar{1}02$ (2θ=33.43°, ψ=27.79°) to distinguish the VO_2 out-of-plane orientation. Supplementary Figure 5(c) (upper panel) shows the Φ scan of VO_2 011 revealing a six fold symmetry, while no peak was observed for the VO_2 $\bar{1}02$ Φ scan (not shown), demonstrating that the film peaks of the on-axis 2θ-ω scan correspond to 020 and 040.[6] The Φ scan of VO_2 011 was related to that of Al_2O_3 012 [Supplementary Figure 4(c) (lower panel)]. The following epitaxial relationship was derived: $(010)_{VO2}$ // $(001)_{Al2O3}$ and $[100]_{VO2}$ // $[120]_{Al2O3}$, in agreement with previous reports.[6,7]

Supplementary Note 4 Growth of VO_2 on Silicon

The growth of VO_2 on silicon was challenging because silicon does not provide an epitaxial template for VO_2 growth.[8,9] Supplementary Figure 6(a) shows reflection high energy electron diffraction (RHEED) pattern of the Si (001) substrate prior to deposition. Main diffraction spots from Si were barely visible and only Kikuchi lines were observed, indicating a thin, amorphous native oxide layer on top of the silicon substrate. The RHEED pattern of the 30 nm VO_2 film on silicon is shown in Supplementary Figure 5(b). A ring-shaped diffraction pattern was observed, showing a polycrystalline film due to the lack of an epitaxial template. AFM scans [Supplementary Figure 6(c)] were performed on VO_2 grown on silicon, showing a polycrystalline microstructure with a pronounced surface roughness (rms value 5.1 nm). Supplementary Figure 6(d) shows the temperature dependent resistivity measurement of this film, demonstrating over 2.6 orders of magnitude change in resistivity across the MIT, larger than commonly reported results in literature,[8,10–13] where some of these films had thicknesses up to hundreds of nanometer; and comparable to the carefully optimized result in literature, as well as their follow up paper.[14,15] This smaller magnitude of resistivity change across the MIT of VO_2 films on silicon compared with that on sapphire wafers has been attributed to the high grain boundary (GB) density intrinsic to the polycrystalline VO_2 film,[16] where GBs acted as electron scatters and thus increased the resistivity of metallic state, while oxygen vacancies can form at GBs and decreased the resistivity of low temperature state by increasing the carrier concentration.[17]

Supplementary References

1. Silversmit, G., Depla, D., Poelman, H., Marin, G. B. & De Gryse, R. Determination of the V2p XPS binding energies for different vanadium oxidation states (V5+ to V0+). *J. Electron Spectros. Relat. Phenomena* **135,** 167–175 (2004).

2. Ko, C., Yang, Z. & Ramanathan, S. Work function of vanadium dioxide thin films across the metal-insulator transition and the role of surface nonstoichiometry. *ACS Appl. Mater. Interfaces* **3,** 3396–401 (2011).

3. Sohn, A. *et al.* Evolution of local work function in epitaxial VO 2 thin films spanning the metal-insulator transition. *Appl. Phys. Lett.* **101,** 191605 (2012).

4. Wang, Y. & Zhang, Z. Synthesis and field emission property of VO2 nanorods with a body-centered-cubic structure. *Phys. E Low-Dimensional Syst. Nanostructures* **41,** 548–551 (2009).

5. Gupta, a. *et al.* Semiconductor to metal transition characteristics of VO[sub 2] thin films grown epitaxially on Si (001). *Appl. Phys. Lett.* **95,** 111915 (2009).

6. Chen, C. *et al.* VO2 multidomain heteroepitaxial growth and terahertz transmission modulation. *Appl. Phys. Lett.* **97,** 211905 (2010).

7. Zhou, Y. & Ramanathan, S. Heteroepitaxial VO2 thin films on GaN: Structure and metal-insulator transition characteristics. *J. Appl. Phys.* **112,** 074114 (2012).

8. Youn, D.-H. *et al.* Phase and structural characterization of vanadium oxide films grown on amorphous SiO[sub 2]/Si substrates. *J. Vac. Sci. Technol. A Vacuum, Surfaces, Film.* **22,** 719 (2004).

9. Jian, J., Chen, A., Zhang, W. & Wang, H. Sharp semiconductor-to-metal transition of VO2 thin films on glass substrates. *J. Appl. Phys.* **114,** 244301 (2013).

10. Yuan, N., Li, J., Li, G. & Chen, X. The large modification of phase transition characteristics of VO2 films on SiO2/Si substrates. *Thin Solid Films* **515,** 1275–1279 (2006).

11. Chae, B. G., Youn, D. H., Kim, H. T., Maeng, S. Y. & Kang, K. Y. Fabrication and Electrical Properties of Pure VO2 Phase Films. 5 (2003). at <http://arxiv.org/abs/cond-mat/0311616>

12. Kovács, G. J. *et al.* Effect of the substrate on the insulator–metal transition of vanadium dioxide films. *J. Appl. Phys.* **109,** 063708 (2011).

13. Dumas-Bouchiat, F., Champeaux, C., Catherinot, A., Crunteanu, A. & Blondy, P. Rf-microwave switches based on reversible semiconductor-metal transition of v O2 thin films synthesized by pulsed-laser deposition. *Appl. Phys. Lett.* **91,** 223505 (2007).

14. Ko, C. & Ramanathan, S. Observation of electric field-assisted phase transition in thin film vanadium oxide in a metal-oxide-semiconductor device geometry. *Appl. Phys. Lett.* **93,** 252101 (2008).

15. Savo, S. *et al.* Reconfigurable anisotropy and functional transformations with VO2-based metamaterial electric circuits. *Phys. Rev. B* **91,** 134105 (2015).

16. Narayan, J. & Bhosle, V. M. Phase transition and critical issues in structure-property correlations of vanadium oxide. *J. Appl. Phys.* **100,** 103524 (2006).

17. Appavoo, K. *et al.* Role of defects in the phase transition of VO2 nanoparticles probed by plasmon resonance spectroscopy. *Nano Lett.* **12,** 780–6 (2012).
